# Blood Neurofilament Light Chain as a Potential Biomarker for Central and Peripheral Nervous Toxicity in Rats

**DOI:** 10.1093/toxsci/kfab122

**Published:** 2021-10-22

**Authors:** Tomoya Sano, Yasushi Masuda, Hironobu Yasuno, Tadahiro Shinozawa, Takeshi Watanabe, Masaaki Kakehi

**Affiliations:** 1 Drug Safety Research and Evaluation, Takeda Pharmaceutical Company Limited, Fujisawa, Kanagawa 251-8555, Japan; 2 Drug Metabolism and Pharmacokinetics Research Laboratories, Takeda Pharmaceutical Company Limited, Fujisawa, Kanagawa 251-8555, Japan

**Keywords:** neurofilament light chain, neurotoxicity, rat

## Abstract

Neurotoxicity is a principal concern in nonclinical drug development. However, standardized and universally accepted fluid biomarkers for evaluating neurotoxicity are lacking. Increasing clinical evidence supports the potential use of neurofilament light (NfL) chain as a biomarker of several neurodegenerative diseases; therefore, we investigated changes in the cerebrospinal fluid (CSF) and serum levels of NfL in Sprague Dawley rats treated with central nervous system (CNS) toxicants (trimethyltin [TMT, 10 mg/kg po, single dose], kainic acid [KA, 12 mg/kg sc, single dose], MK-801 [1 mg/kg sc, single dose]), and a peripheral nervous system (PNS) toxicant (pyridoxine, 1200 mg/kg/day for 3 days). Animals were euthanized 1 (day 2), 3 (day 4), or 7 days after administration (day 8). Increased serum NfL was observed in TMT- and KA-treated animals, which indicated neuronal cell death in the brain on days 2, 4, and/or 8. MK-801-treated animals exhibited no changes in the serum and CSF levels of NfL and no histopathological changes in the brain at any time point. Pyridoxine-induced chromatolysis of the dorsal root ganglion on day 2 and degeneration of peripheral nerve fiber on day 4; additionally, serum NfL was increased. A strong correlation was observed between the serum and CSF levels of NfL and brain lesions caused by TMT and KA, indicating that NfL could be a useful biomarker for detecting CNS toxicity. Additionally, PNS changes were correlated with serum NfL levels. Therefore, serum NfL could serve as a useful peripheral biomarker for detecting both CNS and PNS toxicity in rats.

Central nervous system (CNS) toxicity is a principal concern in drug development because it accounts for approximately 25% of attrition in drug development (Walker *et al.*, 2018) and is a common reason for drug withdrawal and discontinuation in the United States and Europe (Siramshetty *et al.*, 2016). Neurotoxicity has been assessed preclinically using multiple datasets of functional assessments, such as neurobehavioral evaluation and electroencephalogram, coupled with magnetic resonance imaging, histopathology and/or some fluidic biomarkers (Walker *et al.*, 2018). Over the past decade, several neuronal and glial proteins, sphingolipids, and brain-enriched miRNAs have been proposed as potential peripheral neurotoxicity biomarkers (Glushakova *et al.*, 2012; Imam *et al.*, 2018; Ogata *et al.*, 2015). However, there are no standardized and universally accepted fluid biomarkers for evaluating neurotoxicity in nonclinical studies. Thus, the development of novel translatable biomarkers of neurotoxicity will have a beneficial impact not only on nonclinical drug development but also on patient safety in clinical trials.

Neurofilaments are the major component of the axonal cytoskeleton and consist of 3 types of proteins: neurofilament light (NfL), neurofilament medium, and neurofilament heavy chains (Khalil *et al.*, 2018). Neurofilaments are released into the extracellular space when an axon is damaged and subsequently into the cerebrospinal fluid (CSF) and blood (Khalil *et al.*, 2018). Recently, the development of an extremely sensitive and reliable assay based on single-molecule array technology (Simoa) has enabled reliable measurement of blood NfL levels (Khalil *et al.*, 2018; Kuhle *et al.*, 2016). NfL is a novel biomarker, as well as a clinical marker, reflecting disease severity, progression, or therapy response in several neurological disorders such as multiple sclerosis, amyotrophic lateral sclerosis, Huntington’s disease, and Alzheimer’s disease (Disanto *et al.*, 2017; Fyfe, 2019; Gaetani *et al.*, 2019; Kuhle *et al.*, 2019; Olsson *et al.*, 2019; Rodrigues *et al.*, 2020). NfL was recently reported to be a useful biomarker of axonal degeneration in chemotherapy-induced peripheral neurotoxicity in both clinical and nonclinical studies (Kim *et al.*, 2020; Meregalli *et al.*, 2018, 2020), including in a rat model of vincristine/cisplatin/paclitaxel-induced peripheral neurotoxicity (Meregalli *et al.*, 2018, 2020). Additionally, researchers have reported that NfL is elevated in mouse models of Huntington’s disease, amyotrophic lateral sclerosis, Parkinson’s disease, Alzheimer’s disease, and Gaucher disease and that both CSF and blood levels of NfL are convincing and useful markers for monitoring disease progression and severity (Bacioglu *et al.*, 2016; Loeffler *et al.*, 2020; Soylu-Kucharz *et al.*, 2017). Additionally, elevation of NfL levels in the CSF and serum was observed in experimental pneumococcal meningitis in rats (Le *et al.*, 2020). However, little is known regarding the usefulness of NfL as a nonclinical biomarker of CNS toxicity, especially neuronal cell death.

In this study, we employed the Simoa assay to measure serum and CSF NfL levels, evaluated the potential of NfL as a peripheral biomarker of CNS toxicity, and compared the sensitivities of typical neurotoxicity endpoints such as nervous symptoms and histopathology in rat neurotoxicity models induced by trimethyltin (TMT), kainic acid (KA), and MK-801. TMT induces injury in CNS neurons and several nervous symptoms (Balaban *et al.*, 1988; Bushnell and Evans, 1985; Ceccariglia *et al.*, 2020; Crofton *et al.*, 1990; Ogata *et al.*, 2015). KA, an agonist of kainate glutamate receptor subtypes, causes glutamate excitotoxicity in rodents (Zheng *et al.*, 2011), and KA treatment is one of the most reliable models for temporal lobe epilepsy (Bertoglio *et al.*, 2017; Levesque and Avoli, 2013; Zheng *et al.*, 2011). MK-801, an NMDA receptor antagonist (Olney *et al.*, 1989), has been reported to induce temporal neuronal cell vacuolation and neuronal cell death in rats (Fix *et al.*, 1993, 1995; Kuroda *et al.*, 2015). In addition to these CNS toxicants, we measured NfL levels in a pyridoxine-induced peripheral neuropathy model (Jortner, 2000; Krinke and Fitzgerald, 1988).

This study aimed to investigate whether increased NfL levels in the CSF and serum can be utilized for detecting neuronal damage in TMT-, KA-, MK-801-, and pyridoxine-induced nervous toxicity models and to discuss the feasibility and usability of NfL as a blood biomarker of neurotoxicity.

## MATERIALS AND METHODS

###  

####  

##### Animals and treatments

This study was approved by the Institutional Animal Care and Use Committee, Shonan Health Innovation Park. We used male Sprague Dawley rats (Charles River Laboratories Japan Inc.) with weights ranging from 207 to 260 g. The rats were selected using standardized normal values calculated from the body weights. These animals were allocated to 12 groups, each comprising 4 males: 5 groups (Control, TMT, KA, MK-801, and pyridoxine) necropsied at 1 day after the first dose (day 2), 5 groups (Control, TMT, KA, MK-801, and pyridoxine) necropsied at 3 days after the first dose (day 4), and 2 groups (Control and TMT) necropsied at 7 days after the first dose (day 8). The animals were individually housed in metal cages with stainless-steel wire mesh bottoms equipped with a stainless-steel resting board and chew toys (I chew, ASAP and Nylon Bone, Bio Serv., New Jersey) as animal enrichment devices. The conditions of the room were as follows: temperature of control range: 20–26°C, relative humidity of control range: 40–70%, air exchange 10–25 times/h, and a 12-h light/dark cycle. The animals were allowed free access to a pelleted laboratory animal diet (CR-LPF, ORIENTAL YEAST CO. LTD., Tokyo, Japan) and tap water. Animals received a single oral gavage dose of TMT (Tokyo Chemical Industry Co., Ltd., Tokyo, Japan) at 10 mg/kg, a single subcutaneous injection of KA (FUJIFILM Wako Pure Chemical Corporation, Osaka, Japan) at 12 mg/kg, single subcutaneous injection of MK-801 (FUJIFILM Wako Pure Chemical Corporation) at 1 mg/kg or pyridoxine (Sigma-Aldrich Japan, Tokyo, Japan) at 1200 mg/kg/day (600 mg/kg BID) for 1 or 3 days; they were sacrificed at various time points (1, 3, or 7 days after administration). The dose level of each compound was set on the basis of doses reported in the literature (Covolan and Mello, 2000; Fix *et al.*, 1995; Jortner, 2000; Kuroda *et al.*, 2015; Ogata *et al.*, 2015) or internal data (data not presented). Clinical signs were observed daily, and body weight was measured on days 2, 4, and 8.

##### Tissue collection, blood, and CSF sample collection and preparation

For collecting the brain tissues, blood, and CSF samples at each necropsied time point, the animals were anesthetized with 4% isoflurane in oxygen as a carrier gas. Blood samples were collected from the abdominal aorta using a syringe without anticoagulant under isoflurane anesthesia prior to necropsy. The blood samples were kept at room temperature for 30 min and subsequently centrifuged at 1700 × g for 10 min at 4°C for obtaining serum. Immediately after euthanasia by exsanguination, the skin and muscles around the back of the neck were removed, and the dura mater of the cisterna magna was exposed. CSF samples were collected via cisterna magna puncture using a syringe and needle. We necropsied the animals; collected the tissues, including the brain, spinal cord (cervical, thoracic, and lumbar spine), sciatic nerve, tibial nerve, dorsal root ganglion (DRG; including the lumbar spine L1 and L2 and the cervical spine), and eyes (including the optic nerve); and fixed them in 10 vol% neutral buffered formalin for subsequent microscopic examination.

##### Histopathological examination

In accordance with the STP position paper (Bolon *et al.*, 2013), the brain and spinal cord (cervical, thoracic, and lumbar, transverse only) were trimmed, embedded in paraffin, and sectioned. The 4-µm thick sections were stained with hematoxylin and eosin (HE). Additionally, the HE-stained slides of the sciatic nerve, tibial nerve, DRG (including the L1 and L2 segments of the lumbar spine and C1 and C2 segments of the cervical spine), and eyes (including the optic nerve) were prepared for microscopic examination. Staining with Fluoro-Jade C (FJ-C), a neuronal cell death marker, was additionally performed for the brains of representative animals necropsied on days 4 and 8 in the TMT groups and on days 2 and 4 in the KA groups and for the brains of all animals in which neuronal cell necrosis was not identified in HE sections from the other groups. For immunohistochemistry, brain sections of 4 control animals necropsied on day 2 and that of all animals from the TMT and KA groups were prepared for characterizing gliosis observed in the HE sections. After deparaffinization, immunohistochemistry using antiglial fibrillary acidic protein (GFAP) antibody and anti-ionized calcium-binding adapter molecule 1 (Iba1) antibody was performed using Leica Bond-Rx autostainers (Leica Biosystems K.K., Tokyo, Japan) in accordance with the manufacturer’s instructions. Additionally, we performed heat-induced epitope retrieval in BOND Epitope Retrieval Solution 1 (AR9961, Leica Biosystems K.K.) for both antibodies, Bond Polymer Refine Detection (DS-9800, Leica Biosystems K.K.), and blocking using Protein Block (X0909, DAKO, Agilent Technologies Japan, Ltd., Tokyo, Japan) in accordance with standard procedures. The sections were incubated with anti-GFAP rabbit polyclonal antibody (1:2 dilution, IR524, DAKO) or anti-Iba1 rabbit polyclonal antibody (1:5000 dilution, ab178847, Abcam, Cambridge, UK) for 30 min at room temperature. Immunoreactivity was detected and visualized using Bond DAB Enhancer (AR9432, Leica Biosystems K.K.) before the sections were counterstained with hematoxylin.

##### Measurement of NfL levels in CSF and serum

NfL levels in CSF and serum were measured using a Simoa SR-X analyzer (Quanterix Corporation, Massachusetts) with a Simoa NF-light Advantage (SR-X) Kit (Quanterix, No. 103400) according to the manufacturer’s instructions.

##### Statistical analysis

The data on body weights and organ weights were tested by the *F* test for homogeneity of variance between the control group and each test article group. When the variances were homogeneous, Student’s *t* test was used; when the variances were heterogeneous, the Aspin and Welch *t* test was performed to compare the mean in the control group with that in the test article group. Correlations between CSF and serum NfL levels were determined using Pearson’s correlation coefficient.

The *F* test was conducted at a significance level of 0.20, and the other tests were conducted at 2-tailed significance levels of 0.05 and 0.01. Analyses were performed using SAS version 9.3 (SAS Institute Inc.).

## RESULTS

###  

#### Clinical Signs, Body Weight, and Brain Weight

The clinical signs, body weight, and brain weight changes are summarized in Table  1. After a single administration of TMT, convulsions, tremors, twitches, and/or irritability were observed on day 4 and thereafter (day 8). In the KA group, wet dog shakes, twitch, salivation, and/or irritability were noted from the day of dosing (day 1) to day 4. The animals in the MK-801 group showed a severe decrease in locomotor activity on day 1. In the TMT-treatment group, the body weight significantly decreased on days 4 and 8. Additionally, the KA-treated animals showed a reduction in body weight on days 2 and 4. The pyridoxine group showed a decrease in body weight on days 2 and 4, without any neuronal clinical signs. Absolute brain weight did not change in any group on days 2 and 4. On day 8, absolute brain weight was decreased in the TMT group; however, this was possibly related to body weight reduction and animal growth retardation.

**Table 1.** Effects of TMT, KA, MK-801, and Pyridoxine Treatment on Body Weights, Brain Weights, and Nervous Symptoms

Test Article	Trimethyltin				Kainic Acid		MK-801		Pyridoxine	
Dose	10 mg/kg, po, single				12 mg/kg, sc, single		1 mg/kg, sc, single		1200 mg/kg/day, ip, 3 days	
Necropsy timing (day 1 is the day of first dosing)	Day 2	Day 4	Day 8		Day 2	Day 4	Day 2	Day 4	Day 2	Day 4
No. of animals	4	4	4		4	4	4	4	4	4
Body weight (% differ ence from control)	—	−10%*	−13%*		−16%**	−16%*	—	—	−3%	−8%
Absolute brain weight (% difference from control)	—	—	−6%*		—	—	—	—	—	—
Representative clinical signs of potential effects on central or peripheral nerve (incidence and observation timing)										
Decreased locomotor activity	—	—	—	1		—	4	4	—	—
				(day 2)			(1-h AD)			
Prone position	—	—	—	1		—	4	4	—	—
				(day 2)			(1-h AD)			
Hunchback position	—	—	—	1		—	—	—	—	—
				(day 2)						
Bizarre behavior	—	—	—	—		—	—	1	—	—
							(1-h AD)			
Soiled fur	—	—	2	3		1	—	—	—	—
			(days 6–8)	(days 2–4)						
Irritability	—	3	4	4		4	1	4	—	—
		(days 4–8)		(4-h AD, day 2–4)			(1-h AD)			
Salivation	—	—	—	4		3	—	2	—	—
				(1- and 4-h AD)				(1-h AD)		
Irregular respiration	—	1	—	—		—	—	—	—	—
		(4-h AD)								
Staggering gait	—	—	—	1		—	1	—	—	—
				(1-h AD)			(4-h AD)			
Twitch	—	3	4	1		4	—	—	—	—
		(days 4–8)		(4-h AD)						
Tremor	—	—	1	—		—	—	—	—	—
			(days 7 and 8)							
Wet dog shake	—	—	—	4		4	—	—	—	—
				(1-h AD)						
Convulsion	—	—	3	—		—	—	—	—	—
			(days 4, 5, and 7)							

AD, after the first dose; —, not remarkable finding.

* *p* < .05 versus control, ** *p* < .01 versus control.

#### Histopathological Examination

**Figure 1.**
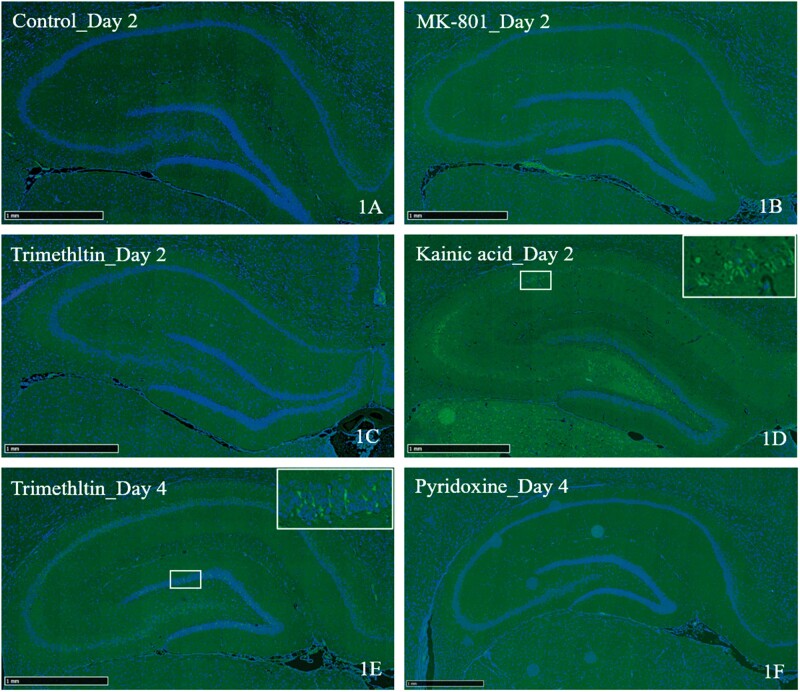
Representative photographs of Fluoro-Jade staining (green) counterstained with nucleus staining (DAPI, blue) in the hippocampus of control rats and rats treated with trimethyltin (TMT), MK-801, Kainic acid (KA), and pyridoxine (bars: 1 mm). Fluoro-Jade-positive neurons of the hippocampus and dentate gyrus are presented in (D; KA_Day 2) and (E; TMT_Day 4). The control_Day 2 (A), MK-801_Day 2 (B), TMT_Day 2 (C), and Pyridoxine_Day 4 (F) samples show no positive staining in the brain. Inset: higher magnification of (D) and (E). Day 2, 1 day after the first dosing; day 4, 3 days after the first dosing.

**Figure 2.**
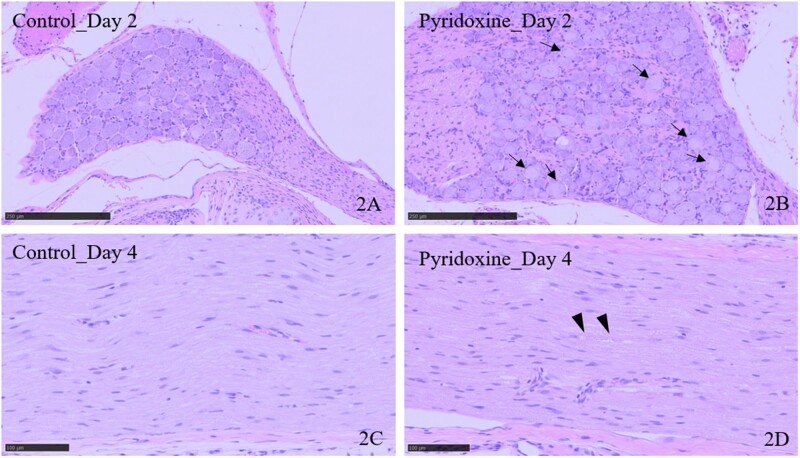
Representative photographs of the dorsal root ganglion (DRG) and peripheral nerve in control and pyridoxine-treated rats (bars: 100 µm). Arrows indicate chromatolysis in the DRG. Arrowhead indicates nerve fiber degeneration in the sciatic nerve. Day 2, 1 day after the first dosing; day 4, 3 days after the first dosing.

Representative histopathological findings in the brain are summarized in Table  2 and Figures  1 and 2.

**Table 2.** Results of Histopathology in TMT, KA, MK-801, and Pyridoxine-Treated Animals

Test article	TMT			KA		MK-801		Pyridoxine	
Dose	10 mg/kg, po, single			12 mg/kg, sc, single		1 mg/kg, sc, single		1200 mg/kg/day, ip, 3 days	
Necropsy timing (day 1 is the day of first dosing)	Day 2	Day 4	Day 8	Day 2	Day 4	Day 2	Day 4	Day 2	Day 4
No. of animals examined	4	4	4	4	4	4	4	4	4
Representative histopathological findings (incidence and severity)									
Cerebrum/necrosis of nerve cell									
Hippocampus	—	4: +	3: +, 1: ++	4: +	3: +, 1: ++	—	—	—	—
Amygdala	—	—	—	4: ++	1: ±, 3:+	—	—	—	—
Thalamus	—	—	2: ±	3: ±, 1:+	3: ±	—	—	—	—
Hypothalamus	—	—	—	2: ±, 1:+	2: ±	—	—	—	—
Entorhinal cortex	—	3: ±	4: ++	4: ++	1: ±-, 3:++	—	—	—	—
Piriform cortex	—	3: ±, 1:+	4: ++	4: ++	1: +, 2:++	—	—	—	—
Septal nucleus	—	2: ±	2: ±	4: +	2: ±, 2:+	—	—	—	—
Retrosplenial cortex	—	—	4: ±	—	—	—	—	—	—
Olfactory bulb	—	1: ±	4: ±	3: ±	3: ±	—	—	—	—
Cellebellum/necrosis of Purkinje cell	—	2: ±	2: ±	—	—	—	—	—	—
DRG/chromatolysis	—	—	—	—	—	—	—	4: ±	4: ±
Dorsal nerve root/degeneration of nerve fiber	—	—	3: ±	—	—	—	—	—	—
Sciatic and tibial nerve/degeneration of nerve fiber	—	—	4: ±	—	—	—	—	—	1: ±

AD, after the first dose; —, no change or not remarkable finding; ±, minimal; +, mild; ++, moderate.

##### TMT group

Neuronal cell necrosis was observed in the olfactory bulb, hippocampus, entorhinal cortex, pyriform cortex, septal nucleus in the cerebrum, and Purkinje cells in the cerebellum from day 4. On day 8, brain lesions were more severe than on day 4, and they expanded to the retrosplenial cortex and thalamus. The necrotic areas were accompanied by increased Iba-1 positive cells, suggesting the occurrence of microgliosis on day 4; on day 8, mixed gliosis identified by Iba-1- and GFAP-positive cells was observed in the brain. Additionally, degeneration of nerve fibers was observed in the dorsal nerve root, tibial nerve, and sciatic nerve on day 8. On day 2, no histopathological or immunohistochemical findings were noted in any of the CNS and peripheral nervous system (PNS) tissues. Additionally, no FJ-C-positive neurons were observed on day 2.

##### KA group

Neuronal cell necrosis was observed in the hippocampus, hypothalamus, thalamus, amygdala, entorhinal cortex, piriform cortex, septal nucleus, and olfactory bulb on days 1 and 4. The necrotic areas were accompanied by increased Iba-1-and GFAP-positive cells, suggesting that mixed gliosis occurred on days 1 and 4.

##### MK-801 group

No histopathological findings were observed in any of the CNS or PNS tissues. The brains of all MK-801-treated animals were negative for FJ-C staining, suggesting a lack of neurodegenerative changes in the examined regions.

##### Pyridoxine group

No pyridoxine-related histopathological findings were observed in the brain or spinal cord; though spontaneous focal necrosis was unilaterally noted in the cerebral cortex of 1 animal on day 2. Neuronal chromatolysis in the DRG was observed on days 2 and 4. Additionally, degeneration of the nerve fibers in the sciatic and tibial nerves was noted in 1 animal on day 4. The brains of all pyridoxine-treated animals were negative for FJ-C staining except for 1 case of spontaneous change, suggesting a lack of pyridoxine-related neurodegenerative changes in the examined regions.

#### NfL Levels in the CSF and Serum

**Figure 3.**
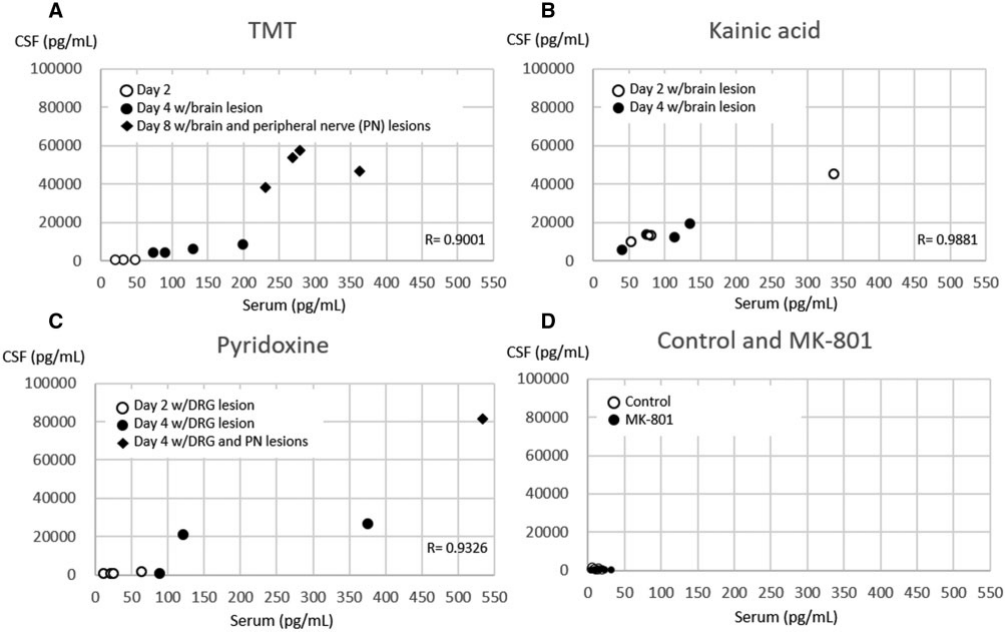
Scatter dot plot graphs of CSF and serum neurofilament light levels for each compound.

The NfL levels in the CSF and serum of the treated animals are shown in Table  3 and Figure  3.

**Table 3.** The Levels of NfL in CSF and Serum Following TMT, KA, MK-801, and Pyridoxine Administration

		CSF (pg/ml)			Serum (pg/ml)	
Test Article	Day 2	Day 4	Day 8	Day 2	Day 4	Day 8
Control	370 ± 171	388 ± 70	816 ± 552	10.3 ± 6.46	8.11 ± 2.27	10.6 ± 4.24
TMT	520 ± 100	5777 ± 2065*	48 897 ± 8531**	32.0 ± 11.85*	123.0 ± 55.75*	285 ± 55.6**
KA	20 273 ± 16 532	12 649 ± 5708*	NA	136 ± 134.32	91.7 ± 41.43*	NA
MK-801	434 ± 186	287 ± 30	NA	20.1 ± 9.41	9.89 ± 4.04	NA
Pyridoxine	717 ± 432	32 354 ± 34 453	NA	30.8 ± 23.18	280.2 ± 212.0	NA

NA, not appricable.

* *p* < .05 versus control, ** *p* < .01 versus control.

The serum/CSF NfL levels and the distribution/severity of histopathological neuronal cell necrosis in the brain were correlated in this study (Supplementary Table 1).

##### TMT group

High NfL levels in serum and CSF were observed in all animals that showed brain lesions on days 4 and 8. The apparent and statistically significant elevation of NfL levels in the serum and CSF were confirmed on day 4 (*p* < .05, approximately 15-fold increase in both CSF and serum) and day 8 (*p* < .01 in both CSF and serum; CSF: approximately 60- and 26-fold increase in the CSF and serum, respectively). TMT-treated animals showed a strong correlation between CSF and serum NfL levels (Figure  3A, *p* < .001, *r* = 0.9001). In particular, the NfL levels in TMT-treated animals on day 8 were much higher than that on day 4, indicating a correlation between NfL levels and the severity of nervous histopathological findings in the CNS and PNS. Despite the lack of histopathological findings in the nerve tissues and NfL levels in the CSF, a statistically significant increase in serum NfL levels was observed in TMT-treated animals on day 2.

##### KA group

Apparent increases in NfL levels in CSF and serum were observed on day 2 (approximately 55- and 13-fold increase in the CSF and serum, respectively) and day 4 (*p* < .05 in both CSF and serum, approximately 33- and 11-fold increase in the CSF and serum, respectively) in all KA-treated animals with brain lesions. NfL levels in the CSF and serum were highly correlated in the KA-treated animals (Figure  3B, *p* < .001, *r* = 0.9881).

##### MK-801 group

No significant differences in NfL levels in the CSF and serum were observed in MK-801-treated animals without any brain lesions.

##### Pyridoxine group

Apparent increases in NfL levels in the CSF and serum were observed in pyridoxine-treated animals with chromatolysis of the DRG and/or nerve fiber degeneration on day 4 (CSF: approximately 83 folds, serum: approximately 35 folds), but not on day 2, when only chromatolysis was observed in the DRG. In the individual data, 1 animal with DRG lesion on days 2 and 4 showed high NfL levels only in the serum (Figure  3C). The levels of NfL in both CSF and serum of pyridoxine-treated animals were highly correlated (Figure  3C, *p* < .001, *r* = 0.9326).

## DISCUSSION

In this study, we demonstrated that both CSF and serum NfL could serve as potential biomarkers for the detection of toxicity in both the CNS and PNS in TMT-, KA-, and pyridoxine-induced rat models of neurotoxicity.

In this study, NfL levels and brain lesions were correlated at various time points in animals treated with TMT and KA (Supplementary Table 1). Increased NfL levels in the serum and CSF were observed in the KA groups on days 2 and 4 and in the TMT groups on days 4 and 8, where neuronal cell death was present in the brain. The NfL level in the TMT group on day 8 was higher than that on day 4. The increased NfL level in the TMT group on day 8 reflected the histopathological progression of the brain lesions by day 8. In the TMT group, increased serum NfL level was also observed on day 2, without any histopathological changes in the brain or alteration of NfL levels in the CSF, suggesting that increased serum NfL levels were not derived from the CNS. However, we cannot deny the possibility that the increase in serum NfL is related to the presence of a very small number of necrotized neuronal cells in the brain. Additionally, peripheral nerve effects such as vacuolation of the spiral ganglion cells in the organ of Corti were reported to be induced 24 h after TMT dosing in rats (Chang and Dyer, 1983). Therefore, further studies involving the quantitative evaluation of neuronal cell death in the brain through stereological analysis or more detailed assessment of peripheral nerve tissues will be needed to clarify not only the precise relationship between the total number of neuronal cell deaths in the whole brain and the NfL values but also the significance of the change in serum NfL in the TMT group on day 2.

In the past decade, multiple fluid biomarker candidates have been proposed for detecting neurotoxicity in nonclinical studies. In KA-treated rats with brain lesions, glial fibrillary acidic protein (GFAP) and ubiquitin C-terminal hydrolase-1 (UCHL-1) were increased in the CSF and brain tissues 24 h after dosing (Glushakova *et al.*, 2012). Peak elevation of UCHL-1 was observed at 6 h after dosing when no brain lesions were confirmed; it might serve as an early marker of neurotoxicity. Although GFAP and UCHL-1 were not detected in the CSF at 48 and 72 h after KA dosing, neuron-specific αII-spectrin breakdown products in the CSF were increased at the same time point (Glushakova *et al.*, 2012). Thus, the panel use of these markers in the CSF was necessary for the assessment of neuronal damage at each time point. Other studies showed that changes in specific miRNAs and metabolomics data in the CSF, blood, and urine were associated with brain lesions in the TMT-induced rat neurotoxicity model (Imam *et al.*, 2018; Ogata *et al.*, 2015). Ogata *et al.* (2015) reported that miR9a-3p and miR384-5p could detect neurodegenerative changes in the rat brain; however, histopathological examination was more sensitive for the early detection of brain lesions. Imam *et al.* (2018) demonstrated that increased acylcarnitine levels in the CSF, plasma, and urine might act as a predictor of neuronal cell death; however, an increase in acylcarnitine was observed transiently before the neuronal lesions. In contrast, the CSF and serum levels of NfL in the TMT- and KA groups were consistently increased in animals with neuronal cell necrosis in the brain, potentially representing a robust marker for detecting acute neuronal cell necrosis with high accuracy. Although the change in NfL levels in the CSF was higher than that in the serum in the TMT and KA groups, the change in NfL levels in the serum was sufficient for detecting brain lesions. Collectively, the present results suggest that NfL is released in the CSF and serum in parallel with neuronal cell damage in the brain, confirming the close relationship between the 2 fluid compartments. To the best of our knowledge, this is the first report to demonstrate the value of serum NfL as a peripheral biomarker of neuronal necrosis in the brain. In previous clinical studies, the measurement of serum NfL levels in neurodegenerative diseases enabled the monitoring of ongoing neuronal damage in real time (Disanto *et al.*, 2017; Fyfe, 2019; Gaetani *et al.*, 2019; Kuhle *et al.*, 2019; Olsson *et al.*, 2019). Our data indicate that NfL could be used for detecting drug-induced neuronal cell necrosis in nonclinical studies as well as for monitoring disease progression in patients with neurodegenerative disorder.

For neurotoxicity evaluation, some of the TMT-treated animals with neuronal cell necrosis in the brain and NfL elevation did not show clinical signs on day 4. On the other hand, all animals in the KA-treated group showed neurological clinical signs such as wet dog shake, twitch, and/or irritability on day 1, whereas neuronal cell necrosis in the brain and NfL elevation were noted on days 2 and 4. Hence, NfL may be a good marker for neuronal cell necrosis in the brain, but in terms of neurotoxicity assessment, abnormal clinical signs may appear earlier, as in the case of KA. In this study, we used severe neurotoxicity models, and further evaluation with milder neurotoxic agents could help to understand the true detectability of neuronal cell necrosis. Additionally, whether NfL can predict neuronal necrosis in the brain or detect biochemical and/or physiological events prior to the obvious neuronal cellular damage was not confirmed in this study, so that further investigations are needed to clarify this point.

NfL levels in both CSF and serum were increased with pyridoxine treatment, a PNS toxicant, on day 4, when nerve fiber degeneration occurred in peripheral nerves. According to previous studies, the primary target of pyridoxine-induced sensory neuropathy in rats is believed to be the cell body of DRG neurons, followed by secondary nerve fiber degeneration (Jortner, 2000; Krinke and Fitzgerald, 1988). In a previous report of increased blood NfL levels in vincristine-, paclitaxel-, and cisplatin-induced rat models of peripheral nerve injury (Meregalli *et al.*, 2018, 2020), serum NfL was highly sensitive to primary axonal damage, especially in paclitaxel-treated rats. Therefore, our data are consistent with previous reports (Meregalli *et al.*, 2018, 2020), and NfL is a good indicator for monitoring nerve fiber lesions in the PNS. Interestingly, in the present study, an increase in serum NfL was observed on days 2 and 4 in some animals, which showed chromatolysis in the DRG but not nerve fiber lesions. These data suggest that serum NfL levels are associated with chromatolysis in the DRG. Additionally, NfL levels in the CSF were increased in the pyridoxine model only on day 4 in this study. Given the lack of CNS lesions in the pyridoxine-treated animals, NfL might be released from the neuronal cell body or its surrounding nerve fibers into the CSF through direct communication between the DRG region and CSF in the subarachnoid space (Joukal *et al.*, 2016).

In this study, we demonstrated that the NfL levels in the serum and CSF can indicate neuronal cell death in the brain and changes in peripheral nerves with a sensitivity similar to that of histopathological examination. Considering the strong correlation between the CSF and serum in neuronal cell necrosis in the brain, the current results provide conceptual evidence for the use of serum NfL as a promising peripheral biomarker for the evaluation of neuronal cell death in the brain. Utilization of NfL for neurotoxicity evaluation in drug development could facilitate identification of neurotoxicity risk and expedite the drug development process through achieving a balance between efficacy and safety and reducing the time to patient access for innovative medicines. 

## SUPPLEMENTARY DATA

Supplementary data are available at *Toxicological Sciences* online.

## Supplementary Material

kfab122_Supplementary_Data
